# Development of paediatric quality of inpatient care indicators for low-income countries - A Delphi study

**DOI:** 10.1186/1471-2431-10-90

**Published:** 2010-12-14

**Authors:** Stephen Ntoburi, Andrew Hutchings, Colin Sanderson, James Carpenter, Martin Weber, Mike English

**Affiliations:** 1 Kenya Medical Research Institute/Wellcome Trust Centre for Geographic Medicine Research - Coast, P.O Box 230, Kilifi, Kenya; 2 London School of Hygiene & Tropical Medicine, Health Services Research Unit, London, UK; 3 London School of Hygiene & Tropical Medicine, Medical Statistics Unit, London, UK; 4 Department of Child and Adolescent Health and Development of the World Health Organization (WHO/CAH), Geneva, Switzerland; 5 Nuffield Department of Medicine, Oxford, UK

## Abstract

**Background:**

Indicators of quality of care for children in hospitals in low-income countries have been proposed, but information on their perceived validity and acceptability is lacking.

**Methods:**

Potential indicators representing structural and process aspects of care for six common conditions were selected from existing, largely qualitative WHO assessment tools and guidelines. We employed the Delphi technique, which combines expert opinion and existing scientific information, to assess their perceived validity and acceptability. Panels of experts, one representing an international panel and one a national (Kenyan) panel, were asked to rate the indicators over 3 rounds and 2 rounds respectively according to a variety of attributes.

**Results:**

Based on a *pre-specified *consensus criteria most of the indicators presented to the experts were accepted: 112/137(82%) and 94/133(71%) for the international and local panels respectively. For the other indicators there was no consensus; none were rejected. Most indicators were rated highly on link to outcomes, reliability, relevance, actionability and priority but rated more poorly on feasibility of data collection under routine conditions. There was moderate to substantial agreement between the two panels of experts.

**Conclusions:**

This Delphi study provided evidence for the perceived usefulness of most of a set of measures of quality of hospital care for children proposed for use in low-income countries. However, both international and local experts expressed concerns that data for many process-based indicators may not currently be available. The feasibility of widespread quality assessment and responsiveness of indicators to intervention should be examined as part of continued efforts to improve approaches to informative hospital quality assessment.

## Background

Delivery of good quality health care has considerable potential to reduce childhood deaths in low-income countries where mortality is high [1,2]. However, both anecdotal and empirical evidence suggest that the quality of care offered in many facilities, both primary and referral, is generally poor [3-8]. Valid and reliable performance measures (indicators) can be used to evaluate quality of care and help health workers to improve it [9]. In some high income countries, quality of care measures are increasingly recognized as a priority to help foster improvement and promote accountability, and substantial investments have been made in their development [10-12]. In contrast, relatively little effort has been put into developing such measures for low-income countries [13].

Recently, the WHO revised an earlier tool for assessing the quality of care in first-referral health care facilities (rural or district level hospitals) in developing countries, based on multi-country experience and informed by discussions at a global WHO meeting in Bali in 2007 [14]. The WHO tool is based on the classical quality of care framework involving structure, process and outcome [15]. Amendments can be made to this tool by countries to suit their local needs. Uptake of the tool will probably depend on "buy in" and support from influential persons in each country. Using this tool, structure and processes of care are rated on a simple semi-qualitative scale (the latter based on a convenience sample of 5 cases) making it useful for rapid appraisal of hospitals. However, such scores are of limited value if the aim is to provide an objective comparison between hospitals within a region or within hospitals at different periods - particularly if different observers use the tool.

The debate about which quality measures are best continues [16]. Proponents of process measures argue that they directly measure actions that are within the control of health workers [17]. However, without appropriate drugs and equipment, health workers may be unable to offer the correct care, making structure a limiting factor in quality improvement [18]. Outcome measures may be intuitively the most useful indicators of quality, but their opponents argue that they are subject to considerable confounding and may require large samples for sufficiently precise measurement.

To decide which measures to use, expert judgement, supported by evidence where available, is often used. Experts, however, differ in opinion and a family of consensus methods such as the nominal group technique (NGT), the Delphi technique, and the RAND/UCLA appropriateness method (a hybrid of the former two), are often used to facilitate communication and avoid the negative social influences associated with group processes [19]. This article reports the results of a Delphi study that aimed to determine which measures of quality of admission care for children in first level referral hospitals in low-income countries were widely supported, using explicit methods. Performance measures can be used to assess quality at different levels - clinical, organisation or population. This study considered measures pertinent to the clinical level. Further, the study aimed to identify process measures which could give quantitative measures of performance and performance change. We had a particular focus on the African setting and also examined the likely acceptability of internationally suggested indicators to an influential Kenyan audience. Ethical approval was obtained from the KEMRI National Ethical Review Committee.

## Methods

### Panel selection

It is recommended that expert panels be multidisciplinary and inclusive of individuals from geographically diverse and culturally disparate areas [20,21]. This heterogeneity is thought to bring a wealth of experience and knowledge, and enhance the richness of the discussion. In the current study, two panels of experts were set up. Panel one, the International Panel, consisted of participants predominantly drawn from the 2007 WHO conference on quality of care for children, and members of an informal WHO-linked Paediatric Quality-of-Care email discussion group. Many have published work on quality of care in low-income settings [14]. Panel two, the national panel, consisted of faculty from the Paediatric Department of the University of Nairobi and senior policy-makers in the Kenyan Ministry of Health. The characteristics of the experts are presented in Additional file 1.

### Establishing the scope

Six common childhood topics were chosen for indicator development: malaria, pneumonia, diarrhoea, meningitis, malnutrition, and problems of the sick newborn. These conditions account for over 80% of morbidity and mortality in hospitals in low-income countries in Africa [22], including Kenya [23]. It has been shown that the care provided for these conditions often varies considerably and is of poor quality [3,6,8,24-27]. However, there are affordable and effective treatments for these conditions, defined by international guidelines promoted by the WHO/UNICEF and, in Kenya, by the government [28,29]. These provide a useful quality standard.

From the generic WHO Hospital assessment tool and the associated international [14], evidence-based guidelines [28], researchers (SN, ME) developed a list of potential indicators. The indicators were equally distributed between those based on structure and those based on process of care. The number of process indicators considered for each condition (e.g. pneumonia, malaria etc) was similar. Outcome measures were not considered for the reasons alluded to above.

**Figure 1:**
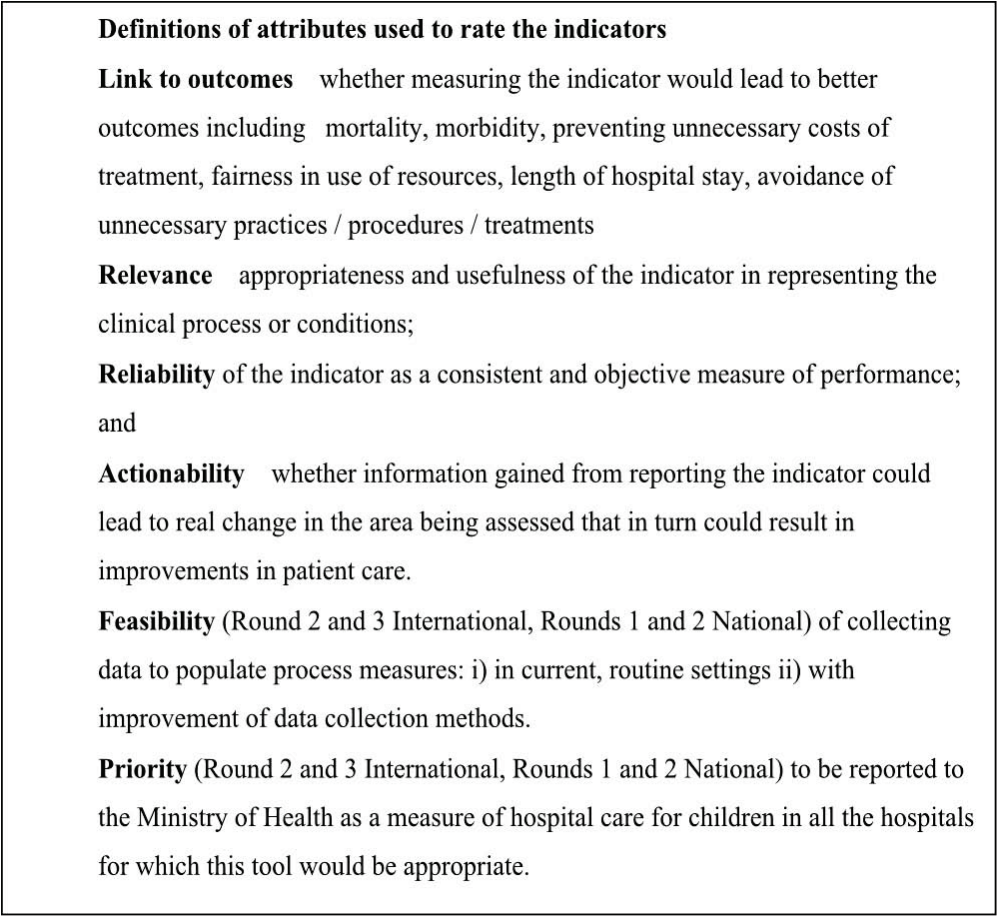
**Definitions of attributes used to rate the indicators**.

A questionnaire was then developed based on the chosen indicators. For all potential indicators the panelists were asked, drawing from their experience, to rate the indicators on various attributes (Figure 1) on a 9 point Likert-type scale ranging from 1 (strongly disagree) to 9 (strongly agree) [30].

Some indicators were composites combining several stage-specific individual indicators. Experts were asked to indicate whether they preferred the composites to the individual constituent indicators. Experts were also asked to indicate in how many areas of the hospital providing paediatric or newborn care a specific item (e.g. a drug or piece of equipment) ought to be present before the item was considered, in aggregate, available at a hospital level.

The limited evidence suggests that better results can be achieved if the participants are given reviews of the literature [30,31]. We did not do this because summarising the evidence would have been an unrealistically large task given the range of topics considered. Moreover, experience suggests that in fact there is often very little high quality evidence for commonly accepted best practices [32]. Instead the experts were provided with both a link to the WHO guidelines for hospital care [28] and to a web-based resource where evidence behind the WHO guidance is progressively being archived http://www.ichrc.org.

### International/WHO expert panel process

The international panel completed three rounds of questionnaires. The first questionnaire was sent in May 2008. This was accompanied by a covering letter giving information about the Delphi process such as the anticipated time required to complete the first questionnaire, how to contact the researchers in case of queries and the deadline for completing the round. In this first round, indicators were rated on only four attributes: link to outcome, reliability, relevance and actionability. Explanations of the attributes were given to the experts and are shown in Figure 1. In addition, experts were asked to suggest new indicators.

In the second round all the experts were provided with their own responses for each indicator and attribute, and the corresponding panel median responses from the first iteration. They were also provided with a summary of the written comments made in the first round complied by one of the investigators (ME) acting as a moderator. Experts were asked to reflect on the feedback and re-rate each item in light of this information. In a few instances, indicator statements were reworded because they were noted by the experts to be ambiguous on the first round. Additional indicators were included following suggestions made in the first round. Two additional attributes were also introduced: i) priority for reporting the indicator to the Ministry of Health and, ii) feasibility of data collection (Figure 1). Opinions on feasibility were requested for process-based indicators only as feasibility of indicator assessment was not considered to be an issue with structural elements - their presence or absence can easily be ascertained. We hoped that these additional attributes, together with the comments from the moderator, would assist the experts to decide which indicators could (feasibility) and should (priority) be included in routine quality assessment. A third round for this panel was conducted similarly. Reminders were regularly sent to the experts (range, 1- 4 reminders) and the process was completed in September 2008.

### National panel process

The local panel of experts completed two rounds of the questionnaire. The experts were invited to the researchers' organization (KEMRI) in June 2008 and the study explained in a Microsoft PowerPoint^® ^presentation. Then the experts completed the questionnaire in private. The international panel Round 2 questionnaire served as the first round for the national panel. The aggregated scores and summary of results from the WHO panel were however not included. The second round was completed 2 weeks later at the same venue, with experts again filling in the questionnaire in private. The questionnaire provided in this round presented the expert's own score and the indicator specific median response of the local panel. Experts were asked to consider revising their previous views in light of this information and the criterion of priority to the Ministry of Health was emphasised. The local expert opinion was used to assess the degree to which recommendations made by an international panel are endorsed in our local setting.

### Analysis

Median scores and frequency of responses in each tertile Likert category (1-3, 4-6, and 7-9) were calculated. We defined an indicator as being accepted with agreement according to the following *pre-specified *criteria (consensus criterion 1) [33]:-

• An indicator was accepted with agreement if two thirds or more of the experts rated the indicator in the upper tertile (7-9) on link to outcomes and a score of 4 or more on reliability, relevance and actionability.

• An indicator was rejected with agreement if two thirds or more of the experts rated the indicator in the lowest tertile (1-3) on link to outcomes.

• An indicator was classified as uncertain/equivocal if they did not fall in either of the above groups.

A second *post hoc *definition of indicator acceptance and agreement, using the same thresholds as above (for consensus criterion 1), but with the further requirement of 'good' agreement about the link to outcomes, defined as an inter-quartile range on the link to outcomes attribute not exceeding two (consensus criterion 2). This latter definition captures the fact that consensus within a group is reflected in smaller variance (smaller IQR) of the responses.

An indicator was defined as a priority for reporting, feasible currently or feasible with improvement if its median score for these specific attribute was 7 or more (consensus criterion 3). A *post hoc *consensus criterion 4 was defined as criterion 3 plus an IQR of less than 2 for these attributes. Finally the process indicators were ranked by ordering them (highest to lowest) using scores achieved on attributes using the following sequence: feasibility with current data, feasibility with improvement of data collection methods and priority for reporting. An indicator was ranked highly if it had high median score on these attributes with a narrow IQR on these attributes. The structure indicators were ranked by priority only. The Wilcoxon signed-rank test was used to test differences in ranking between the two panels. Consensus on the number of places in a hospital where an item must be present to define availability in aggregate was based on a simple majority view (more than 50%) of the experts.

Three-way kappa was used to evaluate reliability of views for the three levels of agreement: accepted, equivocal and rejected. The responses according to consensus criterion 1 from each panel from the final round were treated as responses from two raters for these analyses. The confidence intervals for kappa were obtained using bootstrap methods with 5000 replications. These analyses were carried out in Stata^® ^version 10.2 (StataCorp, Texas, USA) using the *kapci *command and with Stata's pre-recorded weight, *w*, in measuring the importance of disagreements [34]. The kappa values represent the proportionate agreement adjusted for chance and range from 0 (no agreement beyond chance) to 1 (perfect agreement).

## Results

### Responses

Forty percent of those invited to the international panel declined the offer, citing reasons including lack of time and insufficient experience of the topic. 10% and 24 % of the international experts returning round 1 did not complete rounds 2 and 3 respectively. 16/19 of the local experts invited to participate in the process accepted the offer and all but one completed the process.

### Indicator ratings

**Figure 2:**
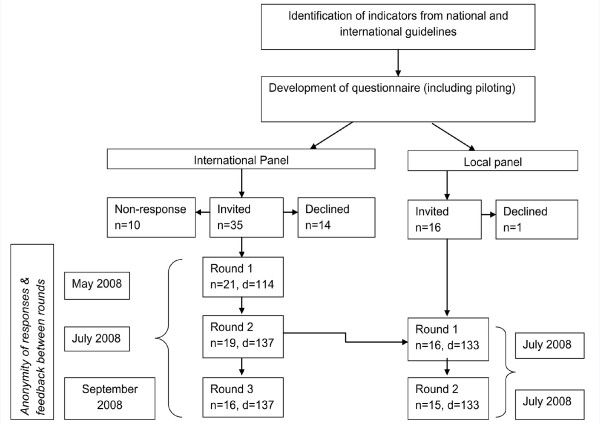
**Procedural flow chart showing the development of quality indicators using the Delphi technique**. n denotes the number of indicators rated in the round. d is the number of experts in the round.

The international experts rated 114 indictors in the first round. A further 23 indicators suggested by them were incorporated and rated in the second round. All 137 indicators were rated in the third round. The local panel rated 133 indicators in both rounds, 132 of which were the same as for the international and local panel (Figure 2). Some indicators suggested by the international panel were not included in the local panel questionnaire and one indicator on HIV was added to the latter's questionnaire after discussion with local policy makers.

**Figure 3:**
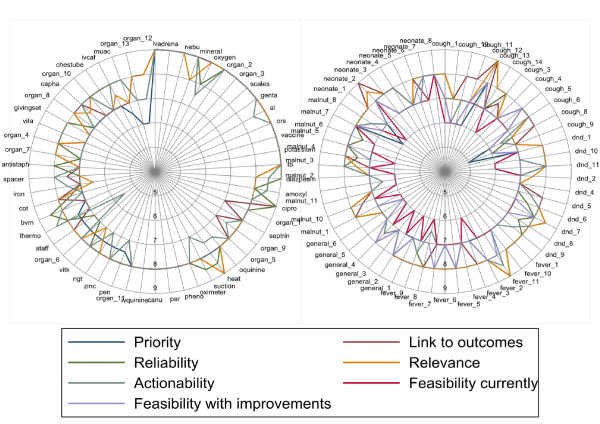
**Spider plots showing the median scores for various attributes as given by the international panel**. The first panel shows the median scores for structure indicators while the second panel shows the scores for process indicators. Labels are used here and the corresponding indicator is described in Additional file 2. The indicators were rated on a 9 point Likert-type scale ranging from 'strongly disagree'(1) to 'strongly agree'(9). Note that the axis begins at five signifying that most indicators were rated highly.

Most indicators were highly rated (median score ≥7) by both panels on the various attributes except on 'feasibility at present', 'feasibility with improvement' and 'priority' (depicted on Figure 3 and further described in Additional file 2). Opinions on what indicators were considered a priority varied more widely (large IQR), particularly within the local panel and for structure indicators. For simplicity and ease of comparison, we consider here only those indicators that were rated by both panels.

The patterns of ratings for each panel were similar between the rounds with only minor changes according to consensus criterion 1 (Table 1). However, the number of indicators accepted in the final round using consensus criterion 2 increased by 79% from the previous round for the international panel but fell by 36% for the local panel, indicating a convergence of views in the final round for the international panel and a divergence of views for the local panel. Conclusions are based on the respective final round ratings which are presented henceforth.

**Table 1:** Changes between rounds in acceptance of indicators (n = 132 indicators)

	International Panel			Local panel		
	**Accepted**	**Equivocal/** **uncertain**	**Rejected**	**Accepted**	**Equivocal/** **uncertain**	**Rejected**

*Round 2/1*						
Criterion 1	110	22	0	98	34	0
Criterion 2	58	74	0	59	73	0

*Final round*						
Criterion 1	111	21	0	93	39	0
Criterion 2	104	28	0	38	94	0

Only indicators rated by both panels are included here.

Based on our *pre-specified *criteria the majority of the indicators (111/132(84.0%)) and 93/132(70.5%) for international and local panels respectively) presented to the experts were accepted. For no indicator was there a consensus for rejection. About a half of those accepted were structure-based - 54/111(48.6%) for the international panel and 43/93(46.2%) for the local panel. When a more strict definition of acceptance and agreement (consensus criterion 2) is imposed, the number of indicators accepted reduces drastically for the local panel (from 93 to 38). There was a much smaller reduction for the international panel (from 111 to 104), reflecting the larger final-round variability in opinions among the local experts. The numbers of indicators accepted in each domain are summarised in Table 2. The top five indicators within each domain accepted by the International panel (consensus criterion 1) and ranked by consensus criterion 3 are listed in Table 3. The full list of indicators is available in Additional file 2 on the journal website.

**Table 2:** Acceptance of care indicators by domain*

	International Panel			Local panel		
	
Domain	Accepted	Equivocal/ uncertain	Rejected	Accepted	Equivocal/ uncertain	Rejected
*Structure:*						
Equipment & drugs (n = 52)	41	11	0	30	22	0
Organisation (n = 14)	13	1	0	13	1	0

*Processes:*						
General (n = 7)	6	1	0	4	3	0
Cough/pneumonia/asthma (n = 15)	12	3	0	10	5	0
Fever/malaria/meningitis (n = 14)	11	3	0	9	5	0
Diarrhoea & dehydration (n = 11)	10	1	0	9	2	0
Malnutrition (n = 11)	10	1	0	10	1	0
Neonatal care (n = 8)	8	0	0	8	0	0

*using consensus criterion 1 and based on final round ratings. Only indicators rated by both panels included here.

**Table 3:** Top five indicators by domain†

Structure: drugs and equipment
1. The availability of intravenous fluids with physiological sodium concentrations (one or more of: Normal saline, Hartmann's solution, or Ringer's Lactate).
2. The availability of Epinephrine (Adrenaline) for injection.
3. The availability of the locally recommended first line oral antimalarial in settings where there is malaria.
4. The availability of Gentamicin.
5. The availability of vaccines including Pentavalent vaccine or DTP or DTP-HepB, BCG polio and measles vaccine in the hospital.

Structure: organisation of care

1. The presence of a system to prioritize severely ill children and group them together for observation in the ward.
2. The presence of a triage system in the outpatients department.
3. The presence of an area in the outpatients section of the hospital that is dedicated to provision of routine and walk-in services for children under 5 years only.
4. Proportion of all admitted children with documentation providing evidence that they were reassessed at least daily (during working days) by a doctor or clinical officer.
5. The availability of up to date hospital records describing paediatric admissions with diagnosis, age, sex and outcomes.

General care for severely ill children:

1. The proportion of children described as unable to feed/drink or with AVPU < V who are prescribed an appropriate fluid or feed regimen.
2. The proportion of children who have documentation that they were clinically assessed (wasting or oedema of Kwashiokor) or assessed with MUAC or WHZ for severe malnutrition.
3. The proportion of admitted children with documentation of assessment for 'danger signs' - these include convulsions, cyanosis, grunting, acidotic breathing, weak pulse, capillary refill, consciouness level (AVPU score) (in)ability to drink, neck stiffness or bulging fontanelle.
4. The proportion of Diazepam prescriptions for convulsions that are of the correct dose (iv or rectal doses according to national guidelines).
5. The proportion of prescriptions for injection Phenobarbitone that are the correct dose.

Cough/pneumoia/asthma:

1. The proportion of children with pneumonia prescribed antibiotics at correct doses (correct dose is defined as weight appropriate dose according to WHO or local guidelines with a ± 20% range of acceptability).
2. The proportion of children prescribed oxygen correctly (including route device and flow rate).
3. The proportion of children with severe or very severe asthma correctly prescribed a steroid (including route of administration dose and frequency).
4. The proportion of children with a diagnosis of pneumonia who are correctly classified as having pneumonia severe pneumonia or very severe pneumonia (The correctness of classification must be based on documentation of appropriate supporting signs).
5. The proportion of children requiring oxygen (i.e. children with cyanosis or other signs of very severe pneumonia/asthma including grunting/head nodding/inability to drink or breastfeed/AVPU < A) actually prescribed oxygen

Fever/malaria/meningitis:

1. The proportion of children with fever in a malaria endemic area who are investigated for malaria (with either a blood slide or rapid test).
2. The proportion of children with malaria prescribed treatment antimalarial that is appropriate to the clinician's classification of severity and local guidelines (correct drug choice dose and frequency)
3. The proportion of children with the presence or absence of fever recorded in their medical notes.
4. The proportion of children with a primary diagnosis of malaria in whom the diagnosis is supported by a positive blood slide/rapid test for malaria.
5. The proportion of children with a diagnosis of meningitis prescribed correct antibiotics (first line appropriate for context or second line if known to have failed prior treatment) in correct doses.

Diarrhoea and dehydration:

1. The proportion of children with dehydration prescribed treatment (including right fluid type volume and rate) that is appropriate to their classification (including shock/severe and some dehydration) and weight.
2. The proportion of children with bloody diarrhoea given correct antibiotic (as recommended by the national guidelines).
3. A composite indicator of the proportion of children assessed completely. This include skin turgor, sunken eyes level of consciousness, ability to drink/breastfeed or sit, capillary refill, peripheral skin temperature and temperature gradient, presence of weak (absent) peripheral pulses, presence of acidotic breathing, irritability, urine output, malnutrition
4. The proportion of diarrhoea cases prescribed Zinc (where this is national policy) at correct dose for age.
5. The proportion of children with diarrhoea who have documentation of whether or not there is blood in the stool.

Malnutrition:

1. The proportion of children in HIV endemic areas with a diagnosis of severe malnutrition who are tested for HIV as an inpatient.
2. The proportion of children with a diagnosis of severe malnutrition prescribed appropriate parenteral antibiotics.
3. The proportion of children with a diagnosis of severe malnutrition receiving Vitamin A on admission.
4. The proportion of children with a diagnosis of severe malnutrition and dehydration prescribed appropriate rehydration fluid (ReSoMal or other low Sodium fluid e.g. half strength Darrows with potassium supplementation).
5. The proportion of children with a diagnosis of severe malnutrition prescribed the correct feed in the recommended volume and frequency.

Neonates:

1. The proportion of sick neonates with a diagnosis of neonatal sepsis prescribed the appropriate antibiotics (including correct choice of drug correct dose for weight and age frequency and route of administration according to guideline).
2. The proportion of babies born in hospital to HIV+ mothers who receive PMTCT therapy in line with national policy.
3. The proportion of babies born in hospital whose mothers have their HIV status known before delivery.
4. The proportion of babies aged <14 days prescribed routine Vitamin K in countries where this is national policy.
5. The proportion of newborn babies who get eye prophylaxis (Tetracycline Eye Ointment).

†using consensus criterion 1 and ranked by consensus criterion 3. Based on final round ratings of the international panel.

Overall, there was very little evidence for a difference in panel rankings for either the structure indicators (z = 0.23, p = 0.81) or the process measures (z = 0.26, p = 0.80). Based on consensus criterion 3, the results for both panels suggest that almost all the structure indicators should be a priority for reporting to the ministry of health while 36/66(54.5%) and 35/66(53.0%) of the process measures were considered both feasible currently and a priority for reporting by the international and local panels respectively (Table 4). Almost all process indicators were considered feasible with improvements in record keeping. However, acceptance rates reduce significantly when dispersion of opinion is considered (consensus criterion 4). In particular the local panel rated significantly fewer indicators as accepted based on priority, feasibility currently or feasibility with improvement when criteria include close consensus.

**Table 4:** Indicators considered a priority or feasible‡

	International	Local	Fisher's exact
	
Consensus criterion 3^§^			
Structure			
Priority	51/54(94.4)	40/43(93.0)	1.00
Process			
Priority	43/57(75.4)	36/50(72.0)	0.83
Feasible currently	38/57(66.7)	43/50(86.0)	0.02
Feasible with improvements	52/57(91.2)	49/50(98.0)	0.21
Structure			
Priority	35/54(64.8)	14/43(32.6)	0.001
Process			
Priority	24/57(42.1)	4/50(8.0)	< 0.001
Feasible currently	27/57(47.4)	5/50(10.0)	< 0.001
Feasible with improvements	43/57(75.4)	7/50(14.0)	< 0.001

§ Consensus criterion 1 requires that indicators have a median score of seven or more on the attribute. || Consensus criterion 2 requires that an indicator has a median score of 7 or more on the attribute and in addition the IQR should be less than 2. Only indicators rated by both panels included here.

Although much more demanding of information, more than half of the international experts expressed a preference for 6 out of the 7 composite indicators presented to them. These composite indicators span multiple processes in managing one case. Local experts preferred 5/7 of these indicators (Additional file 3). However, only a few experts in both panels preferred the proposed composite indicator for children with pneumonia, perhaps thinking it too complex and thus lacking feasibility. Based on simple majority voting, the experts suggested that most of the drugs or equipment needed to be in 2 or more specific areas offering care to children or newborns within the hospital to be considered available at a hospital level (for details see Additional file 4).

For drug doses, 14/15 of international experts and 7/11 local experts who answered the question agreed that a correct dose should be within a range of ± 20% of the dose for weight in the WHO or local guidelines. Using Likert scales we investigated whether the ability of laboratory to perform the following laboratory services was linked to outcomes: blood glucose (bedside); haemoglobin (or full blood count); microscopy or rapid test for malaria (where endemic); HIV testing; blood grouping and cross-matching; and CSF microscopy. All were scored highly (median scores greater than 7) though again opinions varied more for the local panel. Both expert panels tended to rate ability to measure bilirubin lowest (Additional file 3).

For the 132 indicators rated by both panels and based on consensus criterion 1 and final round ratings, the overall raw agreement was 85.9% (kappa 0.85, 95% CI 0.81-0.88). This indicates substantial agreement between the two panels [35]. Agreement varied somewhat by domain but was nonetheless relatively high in all of them (Table 5).

**Table 5:** Agreement between panels' acceptance of care indicators at final round

	Criterion 1		Criterion 2	
	
Domain	Raw agreement (%)	Kappa statistic (95% C1)^¶^	Raw agreement (%)	Kappa statistic (95% C1)^¶^
Structure:				
Equipment & drugs (n = 52)	78.2	0.77(0.70-0.84)	60.3	0.56(0.47-0.64)
Organisation (n = 14)	90.5	0.90(0.77-0.98)	76.2	0.74(0.60-0.87)
*All structure indicators (n = 66)*	*90.9*	*0.90(0.86-0.94)*	*53.5*	*0.54(0.47-0.60)*
Processes:				
General (n = 7)	81.0	0.80(0.61-0.95)	52.4	0.55(0.35-0.76)
Cough/pneumonia/asthma (n = 15)	82.2	0.81(0.67-0.93)	37.8	0.36(0.18-0.48)
Fever/malaria/meningitis (n = 14)	90.5	0.90(0.79-0.98)	57.1	0.57(0.43-0.72)
Diarrhoea & dehydration (n = 11)	93.9	0.94(0.81-1.00)	57.6	0.59(0.42-0.76)
Malnutrition (n = 11) **	1	-	51.5	0.50(0.30-0.68)
Neonatal care (n = 8) **	1	-	75.0	0.74(0.53-0.91)
*All process indicators (n = 66)*	*80.8*	*0.80(0.73-0.86)*	*63.6*	*0.60(0.53-0.68)*
**All indicators (n = 132)**	**85.9**	**0.85(0.81-0.88)**	**58.6**	**0.57(0.51-0.62)**

¶Based on final round ratings and indicators rated by both panels. Kappa 95% CI obtained from bootstrap; ** too few categories of disagreement to calculate kappa and CI correctly.

## Discussion

This study sought to assess the perceived value and validity of a set of indicators of quality of inpatient care proposed for children and newborns admitted to hospital in low-income countries. It used a transparent and inexpensive method that combines scientific evidence and expert opinion. Additionally, we intended to investigate how well recommendations typically issued by international organisations such as the WHO might be accepted by local experts, as such views might influence their local credibility. The Delphi technique proved useful in both respects. The lack of an obligation to meet face to face significantly improved the feasibility of the study as cost was not a constraint on either the size or composition of the international expert panel [36]. Most of the indicators proposed were considered: reliable and relevant to low-income settings; within the capabilities of resource constrained health systems to improve; able to identify areas urgently in need of attention and; to be feasible targets of data collection.

However, one of our anticipated project outputs was identification of a parsimonious set of indicators (about 20-30) that we felt might realistically form the basis of routine and widespread reporting to ministries of health in low-income countries. In this respect it can be argued that the process failed, with support for a large number of indicators of quality of care, making implementation of routine, national quality assessment incorporating all of these potentially more difficult. The demonstrated support for a large number of indicators might be interpreted as an endorsement of the scope and content of the original WHO assessment tool and, by extension, a desire to ensure that any assessment tool for hospital inpatient care should span the resources, assessment tasks and management required to provide effective care for multiple, important diseases. Alternatively it may reflect in part the numerous indicators presented to the experts. Presenting a large initial set of indicators was meant to minimize the risk of missing potentially important issues and prevent a small number of investigators (SN, ME) imposing their priorities at the outset. Interestingly some of the national panel, when prompted to prioritise indicators for reporting to the ministry of health, were more likely than members of the international panel to 'downgrade' indicators (although only to an uncertain status) producing a potentially shorter list and resulting in less apparent consensus in their final round.

It is reassuring that there were high levels of agreement between the local and international panels. Worth noting is the high agreement achieved on the sets of indicators for care of sick neonates and malnourished children. This may have been influenced by increasing recent appreciation globally of the high case fatality rates in these groups. Within panels however, there was somewhat more divergence (larger variance) around structural elements of care perhaps reflecting different experts' experiences of resource environments or the poor scientific evidence available supporting their contribution to better outcomes.

Responses on the feasibility of data collection for assessment of an indicator under current conditions in low-income settings varied, suggesting many panellists were concerned that assessment of many important process indicators requiring data from routine review of medical charts may not be possible currently. There was however optimism that measures could be instituted to improve quality of data. This concern is echoed more generally internationally [37], supported by experience nationally [38,39]. However, an unexpected finding was that indicators for neonatal care were accorded generally higher scores on feasibility. Our experience of working in district hospitals in Kenya [7] and from published data elsewhere [8] seem contrary to these views. It is possible, therefore, that the views expressed by the experts on feasibility are overoptimistic and unduly influenced by their desire to promote certain indicators. Before any indicators are widely adopted they should be tested for feasibility and ability to detect significant change in performance (sensitivity) [40]. We are in the process of evaluating the feasibility of the accepted set of indicators using data from 8 Kenyan district hospitals. More generally, improving the quality of care within a health system will need improvements in information system.

As our goal was to develop a tool that might be routinely used to provide quantitative measures of quality at scale within the capacity of an existing health system, our focus was case record review. However, there are alternative methods of collecting data on the process of care such as direct observation or use of vignettes. Direct observation may influence care, and would be time consuming and costly if a sample size sufficient to produce a quantitative estimate is desired [41]. Vignettes, perhaps best for assessing health worker knowledge, have been used but similarly require organised access to multiple health workers and so may be hard to implement at scale in African settings [42].

There are potential limitations in our study that warrant mention. First, the results reported here represent the opinions of only a few, non-randomly selected individuals. However, our international panel had members with wide and long clinical and quality improvement experience in low-income settings. The local panel consisted largely of experts drawn from an academic referral centre. However, the local experts are influential, frequently called upon by government to provide expert technical advice and are responsible for training of health workers. They thus represent an important constituency in brokering the acceptance or rejection of international recommendations at a country level.

Second, we did not grade or present the strength of evidence linking indicators to outcomes in the questionnaires. This was in part due to the scale and scope of the task with considerable implications on workload for both the researchers and expert panellists. Moreover, the indicators were derived from the practices or resource implications of existing WHO recommendations for hospital care [28]. Third, our definition of consensus, though based on published studies, remains somewhat arbitrary [30,43]. Altering the definition of consensus, for example by calculating mean scores across attributes for each indicator, did not result in significant change in those accepted by the predefined criteria (data not shown). The *post hoc *analysis (consensus criterion 2) may be useful if the intention is to reduce the list of indicators. Fourth, the process did not involve experts meeting face to face. While this reduced the cost, many may argue that an opportunity for experts to meet would have stimulated useful discussion on contentious issues [31]. We did however encourage experts to make comments which we then summarised and fed back to the group through a moderator.

Methodologically, some valuable lessons were learnt. First, Delphi studies are time consuming with an average turnabout time between rounds of approximately 1.5 months for emailed questionnaires. Second, the method of delivering the instructions may have an effect on the results of the process. The local panel received instructions by word of mouth and were more likely to prioritise indicators in round 2 compared to the international experts who were given written instructions. Finally, there is scope for refining definitions of consensus by allowing the participants to decide on an appropriate definition instead of imposing one.

## Conclusions

Measurement of the quality of care is a prerequisite for determining whether quality of care is improving. Although there remains significant challenges in defining such measures, this study represents the first attempt at a transparent, consensus based, international approach to identify indicators of quality hospital care for children and newborns suitable for use in low-income settings. For process based measures, feasibility of data collection remains a concern and should be further evaluated. This study, based on a transparent process, helps formally define widely acceptable, quantitative indicators and provides a platform for further debate and continuing indicator development that should include the review and updating of indicators as priorities and technologies change and interventions to improve quality of care are scaled up. Such reviews may be more likely if a relatively inexpensive process such as the one described here is used.

## Competing interests

The authors declare that they have no competing interests.

## Authors' contributions

SN and ME conceptualized and contributed to the design of this study. ME worked with SN to collect the data. JC, AH, CS and MW participated in the analysis and interpretation of the findings. SN wrote the first draft of the manuscript and all others (including members of the Paediatric Quality of Hospital Care Indicator Panel listed below) reviewed and revised drafts of the manuscript. All authors read and approved the final manuscript.

## Pre-publication history

The pre-publication history for this paper can be accessed here:

http://www.biomedcentral.com/1471-2431/10/90/prepub

## Supplementary Material

Additional file 1 **Characteristics of the experts**. † This represents the proportion of experts who indicated the particular professional category -- experts were allowed to indicate more than one category; N, total number of experts; n experts in the specific category Click here for file

Additional file 2 **List of individual indicators and their ratings**. This excel spreadsheet list all indicators, provides the median scores with interquartile ranges, consensus status and the rank of the indicators for both the international and local panels. Click here for file

Additional file 3 **Composite indicators and other questions**. This excel spreadsheet provides the number of experts supporting a composite indicator and scores given for additional questions mentioned in the text. Click here for file

Additional file 4 **Indications of where an item needs to be in the hospital to be considered available**. The tables show areas where more than 50% of experts indicated that an item ought to be present to be considered available at a hospital level. Click here for file

## References

[B1] English M Child survival: district hospitals and paediatricians Arch Dis Child 2005 90 974 978 10.1136/adc.2005.074468 16113136 PMC1720588

[B2] Duke T Wandi F Jonathan M Matai S Kaupa M Saavu M Subhi R Peel D Improved oxygen systems for childhood pneumonia: a multihospital effectiveness study in Papua New Guinea Lancet 2008 372 9646 1328 1333 10.1016/S0140-6736(08)61164-2 18708248

[B3] Nolan TAP Cunha AJ Muhe L Qazi S Simoes EA Tamburlini G Weber M Pierce NF Quality of hospital care for seriously ill children in less-developed countries The Lancet 2000 357 106 110 10.1016/S0140-6736(00)03542-X 11197397

[B4] Zurovac D Rowe AK Quality of treatment for febrile illness among children at outpatient facilities in sub-Saharan Africa Ann Trop Med Parasitol 2006 100 4 283 296 10.1179/136485906X105633 16762109

[B5] English M Esamai F Wasunna A Were F Ogutu B Wamae A Snow RW Peshu N Delivery of paediatric care at the first-referral level in Kenya Lancet 2004 364 9445 1622 1629 10.1016/S0140-6736(04)17318-2 15519635

[B6] English M Esamai F Wasunna A Were F Ogutu B Wamae A Snow RW Peshu N Assessment of inpatient paediatric care in first referral level hospitals in 13 districts in Kenya Lancet 2004 363 9425 1948 1953 10.1016/S0140-6736(04)16408-8 15194254

[B7] English M Ntoburi S Wagai J Mbindyo P Opiyo N Ayieko P Opondo C Migiro S Wamae A Irimu G An intervention to improve paediatric and newborn care in Kenyan district hospitals: Understanding the context Implement Sci 2009 4 42 10.1186/1748-5908-4-42 19627588 PMC2724481

[B8] Reyburn H Mwakasungula E Chonya S Mtei F Bygbjerg I Poulsen A Olomi R Clinical assessment and treatment in paediatric wards in the north-east of the United Republic of Tanzania Bull World Health Organ 2008 86 2 132 139 10.2471/BLT.07.041723 18297168 PMC2647389

[B9] Massoud R Askov K Reinke J Franco L Bornstein T Knebel E MacAulay C A Modern Paradigm for Improving Healthcare Quality 2001 Bethesda: Quality Assurance Project

[B10] Mainz J Quality indicators: essential for quality improvement Int J Qual Health Care 2004 16 suppl 1 i1 2 10.1093/intqhc/mzh036 15059986

[B11] Marshall M Klazinga N Leatherman S Hardy C Bergmann E Pisco L Mattke S Mainz J OECD Health Care Quality Indicator Project. The expert panel on primary care prevention and health promotion Int J Qual Health Care 2006 18 Suppl 1 21 25 10.1093/intqhc/mzl021 16954512

[B12] McGlynn EA Asch SM Developing a clinical performance measure Am J Prev Med 1998 14 3 Suppl 14 21 10.1016/S0749-3797(97)00032-9 9566932

[B13] Rowe A de Savigny D Lanata C Victora C How can we achieve and maintain high-quality performance of health workers in low-resource settings? Lancet 2005 366 1026 1035 10.1016/S0140-6736(05)67028-6 16168785

[B14] Campbell H Duke T Weber M English M Carai S Tamburlini G Global initiatives for improving hospital care for children: state of the art and future prospects Pediatrics 2008 121 4 e984 992 10.1542/peds.2007-1395 18381526 PMC2655645

[B15] Donabedian D The quality care: How can it be assessed? JAMA 1988 260 12 1743 1748 10.1001/jama.260.12.1743 3045356

[B16] Mant J Process versus outcome indicators in the assessment of quality of health care International Journal for Quality in Health Care 2001 13 475 480 10.1093/intqhc/13.6.475 11769750

[B17] Reerink IH Sauerborn R Quality of primary health care in developing countries: recent experiences and future directions Int J Qual Health Care 1996 8 2 131 139 10.1093/intqhc/8.2.131 8792168

[B18] Gilson L Magomi M Mkangaa E The structural quality of Tanzanian primary health facilities Bull World Health Organ 1995 73 1 105 114 7704920 PMC2486583

[B19] Campbell SM Braspenning J Hutchinson A Marshall M Research methods used in developing and applying quality indicators in primary care Qual Saf Health Care 2002 11 4 358 364 10.1136/qhc.11.4.358 12468698 PMC1758017

[B20] Hutchings A Raine R A systematic review of factors affecting the judgments produced by formal consensus development methods in health care J Health Serv Res Policy 2006 11 3 172 179 10.1258/135581906777641659 16824265

[B21] Fretheim A Schunemann HJ Oxman AD Improving the use of research evidence in guideline development: 3. Group composition and consultation process Health Res Policy Syst 2006 4 15 10.1186/1478-4505-4-15 17134482 PMC1702349

[B22] Black R Morris S Bryce J Where and why are 10 million children dying every year? The Lancet 2003 361 2226 2234 10.1016/S0140-6736(03)13779-8 12842379

[B23] Demographic and Health Survey - Preliminary Report 2009 Nairobi: National Council for Population and Development, Central Bureau of Statistics & Ministry of Planning and National Development, Republic of Kenya

[B24] English M Esamai F Wasunna A Were F Ogutu B Wamae A Snow R Peshu N Delivery of paediatric care at the first-referral level in Kenya The Lancet 2004 364 1622 1629 10.1016/S0140-6736(04)17318-2 15519635

[B25] Reyburn H Mbatia R Drakeley C Carneiro I Mwakasungula E Mwerinde O Saganda K Shao J Kitua A Olomi R Overdiagnosis of malaria in patients with severe febrile illness in Tanzania: a prospective study BMJ 2004 329 7476 1212. 10.1136/bmj.38251.658229.55 15542534 PMC529364

[B26] Chandler CI Mwangi R Mbakilwa H Olomi R Whitty CJ Reyburn H Malaria overdiagnosis: is patient pressure the problem? Health Policy Plan 2008 23 3 170 178 10.1093/heapol/czm046 18321889

[B27] Chandler CI Nadjm B Boniface G Juma K Reyburn H Whitty CJ Assessment of children for acute respiratory infections in hospital outpatients in Tanzania: what drives good practice? Am J Trop Med Hyg 2008 79 6 925 932 19052307

[B28] World Health Organization Hospital care for children: Guidelines for the management of common illnesses with limited resources 2006 Geneva: WHO

[B29] Irimu G Wamae A Wasunna A Were F Ntoburi S Opiyo N Ayieko P Peshu N English M Developing and introducing evidence based clinical practice guidelines for serious illness in Kenya Arch Dis Child 2008 93 9 799 804 10.1136/adc.2007.126508 18719161 PMC2654066

[B30] Murphy MK Black NA Lamping DL McKee CM Sanderson CF Askham J Marteau T Consensus development methods, and their use in clinical guideline development Health Technol Assess 1998 2 3 i iv 1-88 9561895

[B31] Raine R Sanderson C Hutchings A Carter S Larkin K Black N An experimental study of determinants of group judgments in clinical guideline development Lancet 2004 364 9432 429 437 10.1016/S0140-6736(04)16766-4 15288741

[B32] Brewster DR Critical appraisal of the management of severe malnutrition: 1. Epidemiology and treatment guidelines J Paediatr Child Health 2006 42 10 568 574 10.1111/j.1440-1754.2006.00931.x 16972961

[B33] Cantrill JA Sibbald B Buetow S Indicators of the appropriateness of long-term prescribing in general practice in the United Kingdom: consensus development, face and content validity, feasibility, and reliability Qual Health Care 1998 7 3 130 135 10.1136/qshc.7.3.130 10185138 PMC2483608

[B34] Reichenheim ME Confidence intervals for the kappa statistic Stata Journal 2004 4 4 421 428

[B35] Landis JR Koch GG The measurement of observer agreement for categorical data Biometrics 1977 33 1 159 174 10.2307/2529310 843571

[B36] Keeney S Hasson F McKenna H Consulting the oracle: ten lessons from using the Delphi technique in nursing research J Adv Nurs 2006 53 2 205 212 10.1111/j.1365-2648.2006.03716.x 16422719

[B37] AbouZahr C Adjei S Kanchanachitra C From data to policy: good practices and cautionary tales Lancet 2007 369 9566 1039 1046 10.1016/S0140-6736(07)60463-2 17382830

[B38] Okiro E Hay S Gikandi P Sharif S Noor A Peshu N Marsh K Snow R The decline in paediatric malaria admissions on the coast of Kenya Malar J 2007 15 6 151 10.1186/1475-2875-6-151 PMC2194691 18005422

[B39] Gething PW Noor AM Goodman CA Gikandi PW Hay SI Sharif SK Atkinson PM Snow RW Information for decision making from imperfect national data: tracking major changes in health care use in Kenya using geostatistics BMC Med 2007 5 37 10.1186/1741-7015-5-37 18072976 PMC2225405

[B40] Schouten JA Hulscher ME Wollersheim H Braspennning J Kullberg BJ van der Meer JW Grol RP Quality of antibiotic use for lower respiratory tract infections at hospitals: (how) can we measure it? Clin Infect Dis 2005 41 4 450 460 10.1086/431983 16028151

[B41] Rowe AK Lama M Onikpo F Deming MS Health worker perceptions of how being observed influences their practices during consultations with ill children Trop Doct 2002 32 3 166 167 12139161 10.1177/004947550203200317

[B42] Solon O Woo K Quimbo SA Shimkhada R Florentino J Peabody JW A novel method for measuring health care system performance: experience from QIDS in the Philippines Health Policy Plan 2009 24 3 167 174 10.1093/heapol/czp003 19224955 PMC2733796

[B43] Powell C The Delphi technique: myths and realities J Adv Nurs 2003 41 4 376 382 10.1046/j.1365-2648.2003.02537.x 12581103

