# Supplementary material for: Development of paediatric quality of inpatient care indicators for low-income countries - A Delphi study
Source: BMC Pediatr. 2010 Dec 14;10:90. doi: 10.1186/1471-2431-10-90 (PMC3022793; doi:10.1186/1471-2431-10-90)
Supplement: Additional file 1 — Characteristics of the experts. † This represents the proportion of experts who indicated the particular professional category -- experts were allowed to indicate more than one category; N, total number of experts; n experts in the specific category [file 1471-2431-10-90-S1.DOC]

Additional file 1: Characteristics of the experts

|  | International Panel | Local panel |
| --- | --- | --- |
| Number of experts(N) |  |  |
| Invited | 35 | 19 |
| Round 1 | 21 | - |
| Round 2 | 19 | 16 |
| Round 3 | 16 | 15 |
| Age |  |  |
| 31-40 | 2 | 5 |
| 41-50 | 15 | 6 |
| >51 | 4 | 5 |
| Total years of Experience (Median/Range) | 2(8-40) | 20(10-37) |
| Years of experience in Low Income Countries (Median/Range) | 13(1-25) | 20(10-30) |
| Profession n/N (%)† |  |  |
| Practicing Clinical pediatrician | 12/21(57) | 15/16(93) |
| Clinical pediatric research | 12/21(57) | 5/16(31) |
| Professional in Quality Improvement | 1/21(5) | 2/16(13) |
| Public health expert | 4/21(19) | 2/16(13) |
| Health systems research | 3/21(14) | 2/16(13) |
| Employer / employment status(n/N) |  |  |
| Working for government health system | 7/21 | 12/16 |
| Working in private health sector | 1/21 | - |
| Working for UN organization | 4/21 | - |
| Working for public university / academic group | 9/21 | 13/16 |
| Working for non-governmental organization | - | 1/16 |
