# Supplementary material for: Development of paediatric quality of inpatient care indicators for low-income countries - A Delphi study
Source: BMC Pediatr. 2010 Dec 14;10:90. doi: 10.1186/1471-2431-10-90 (PMC3022793; doi:10.1186/1471-2431-10-90)
Supplement: Additional file 4 — Indications of where an item needs to be in the hospital to be considered available. The tables show areas where more than 50% of experts indicated that an item ought to be present to be considered available at a hospital level. [file 1471-2431-10-90-S4.DOC]

Additional file 3

Table 1: Indications of where items should be present (Blue shade =International panel; Grey shades=Local panel)

|  | ***MCH†*** | | ***Casualty¶*** | | ***Labor ward*** | | ***Nursery*** | | ***Paediatric ward*** | | ***All the areas listed*** | |
| --- | --- | --- | --- | --- | --- | --- | --- | --- | --- | --- | --- | --- |
| Working bag valve mask device (BVM) with right size masks |  |  |  |  |  |  |  |  |  |  |  |  |
| Oxygen administration system (source & flow meters(where appropriate) & delivery(prongs, catheters) |  |  |  |  |  |  |  |  |  |  |  |  |
| Working pulse oximeter and probes |  |  |  |  |  |  |  |  |  |  |  |  |
| Suction machine |  |  |  |  |  |  |  |  |  |  |  |  |
| Iv giving sets with chambers for paediatric use |  |  |  |  |  |  |  |  |  |  |  |  |
| Paediatric cannula |  |  |  |  |  |  |  |  |  |  |  |  |
| Intraosseous needle |  |  |  |  |  |  |  |  |  |  |  |  |
| Chest tubes |  |  |  |  |  |  |  |  |  |  |  |  |
| Nebuliser |  |  |  |  |  |  |  |  |  |  |  |  |
| Spacers |  |  |  |  |  |  |  |  |  |  |  |  |
| Stethoscope |  |  |  |  |  |  |  |  |  |  |  |  |
| Weighing scales for infants / children |  |  |  |  |  |  |  |  |  |  |  |  |
| Measuring board |  |  |  |  |  |  |  |  |  |  |  |  |
| Thermometers |  |  |  |  |  |  |  |  |  |  |  |  |
| Heat source for maintaining warmth of very sick newborns / children |  |  |  |  |  |  |  |  |  |  |  |  |
| Torch for throat examination |  |  |  |  |  |  |  |  |  |  |  |  |
| Otoscope |  |  |  |  |  |  |  |  |  |  |  |  |

Table 2: Indications of where drugs need to be present (Blue shade =International panel; Grey shades=Local panel)

|  | ***MCH*** | | ***Casualty*** | | ***Labor ward*** | | ***Nursery*** | | ***Paediatric ward*** | | ***All the areas listed*** | |
| --- | --- | --- | --- | --- | --- | --- | --- | --- | --- | --- | --- | --- |
| Epinephrine |  |  |  |  |  |  |  |  |  |  |  |  |
| Benzyl Penicillin (or Ampicillin) |  |  |  |  |  |  |  |  |  |  |  |  |
| Gentamicin |  |  |  |  |  |  |  |  |  |  |  |  |
| Ceftriaxone |  |  |  |  |  |  |  |  |  |  |  |  |
| Flucloxacillin |  |  |  |  |  |  |  |  |  |  |  |  |
| Chloramphenical IV |  |  |  |  |  |  |  |  |  |  |  |  |
| Chloramphenical oral |  |  |  |  |  |  |  |  |  |  |  |  |
| Oral Co-trimoxazole |  |  |  |  |  |  |  |  |  |  |  |  |
| Oral Metronidazole |  |  |  |  |  |  |  |  |  |  |  |  |
| Oral Amoxicillin |  |  |  |  |  |  |  |  |  |  |  |  |
| Oral Ciprofloxacin / Nalidixic acid |  |  |  |  |  |  |  |  |  |  |  |  |
| IV Diazepam |  |  |  |  |  |  |  |  |  |  |  |  |
| Phenobarbitone injection |  |  |  |  |  |  |  |  |  |  |  |  |
| Oral first line antimalarial |  |  |  |  |  |  |  |  |  |  |  |  |
| Oral second line antimalarial |  |  |  |  |  |  |  |  |  |  |  |  |
| IV first line antimalarial |  |  |  |  |  |  |  |  |  |  |  |  |
| Fluconazole (oral) |  |  |  |  |  |  |  |  |  |  |  |  |
| ORS |  |  |  |  |  |  |  |  |  |  |  |  |
| Ringers lactate (or Normal Saline or Hartmann’s) |  |  |  |  |  |  |  |  |  |  |  |  |
| 10% Dextrose (or 50% and water / 5% to make 10%) |  |  |  |  |  |  |  |  |  |  |  |  |
| Oral potassium supplement |  |  |  |  |  |  |  |  |  |  |  |  |
| Iron tablet/syrup |  |  |  |  |  |  |  |  |  |  |  |  |
| Predinosolone |  |  |  |  |  |  |  |  |  |  |  |  |
| Vitamin A |  |  |  |  |  |  |  |  |  |  |  |  |
| Zinc sulphate |  |  |  |  |  |  |  |  |  |  |  |  |
| Vitamin K injection |  |  |  |  |  |  |  |  |  |  |  |  |
| Mineral mix |  |  |  |  |  |  |  |  |  |  |  |  |
| Anti- TB |  |  |  |  |  |  |  |  |  |  |  |  |
| Vaccines including Pentavalent vaccine or DTP or DTP-Hep B, BCG, polio and measles vaccine |  |  |  |  |  |  |  |  |  |  |  |  |

Footnotes for tables 1 and 2

***†***MCH=Maternal and Child health clinic dedicated to under 5 and pregnant women; ***¶***Casualty includes outpatient departments that are not entirely dedicated to children;

The shaded areas indicate items and areas that had a simple majority support (>50% of experts choosing them);Blue indicates the international expert panel choices while grey indicated local panel choices.
